# A high effective expression of human D-glucuronyl C5-epimerase with dimer structure in *Escherichia coli*

**DOI:** 10.3389/fmicb.2025.1641598

**Published:** 2025-07-31

**Authors:** Qin-xia Song, Li-jian Guo

**Affiliations:** ^1^Key Laboratory of Microbial Resources Exploitation and Application of Gansu Province, Institute of Biology, Gansu Academy of Sciences, Lanzhou, China; ^2^State Key Laboratory of Aridland Crop Science, Gansu Agricultural University, Lanzhou, China

**Keywords:** D-glucuronyl C5-epimerase, heparan sulfate biosynthesis, *Escherichia coli* protein expression, two-step protein purification, dimer structure

## Abstract

**Introduction:**

Heparan sulfate (HS), a linear anionic polysaccharide, participates in many physiological processes and exhibits many pharmacological activities. D-glucuronyl C5-epimerase (Glce) is one of the key enzymes in the biosynthesis of heparan sulfate proteoglycans. However, the recombinant Glce protein exhibits reduced catalytic activity and production yield, which substantially impedes the development of enzymatic methods for producing pharmaceutical-grade heparin.

**Methods:**

In this experiment, we established a valid method for heterologous expression in Escherichia coli (*E. coli*) and subsequent purification of two N-terminal truncated Glce proteins using the SUMO-fused expression system. Characterization of human Glce^167-617^ was described by dynamic light scattering size-exclusion chromatography, and X-ray crystallographic.

**Results:**

In the present study, we successfully overexpressed and purified human Glce^167-617^ protein in *E. coli*. Subsequently, the recombinant Glce^167-617^ was found to exist as a dimer in solution. X-ray crystallographic result further confirmed its dimeric assembly while maintaining the integrity of the catalytic domain.

**Discussion:**

In summary, this study successfully overexpressed and purified human Glce protein in *E. coli*. The purified Glce protein will be applied to chemoenzymatic synthesis of heparin and heparan sulfates in vitro, which facilitating the future bioengineering of pharmaceutical heparins.

## 1 Introduction

Heparan sulfate, a highly sulfated polysaccharide, constitutes a fundamental component of cell surface and extracellular matrix structures (Préchoux et al., 2015). This glycosaminoglycan involves in a variety of biological activities including cell growth, blood coagulation, embryonic development, inflammatory response and tumor metastasis (Bartolini et al., 2020; Bendersky et al., 2020). HS biosynthesis is accomplished through multi-step enzymatic reactions following the polymerization of its precursor disaccharide units. The HS chain precursor is composed of GlcA-GlcNAc repeating disaccharide units. N-deacetylation/N-sulfation of glucosamine is catalyzed by N-deacetylase/N-sulfotransferase (NDST), followed by the epimerization of glucuronic acid (GlcA) unit to L-iduronic acid (IdoA) by Glce protein. The side chain of the polysaccharide further undergoes sulfation modification by 2-O-sulfatetransferase (2OST), 6-O-sulfatetransferase (6OST) and 3-O-sulfate transferase (3OST) (El Masri et al., 2017; Préchoux et al., 2015). In vertebrates, there are multiple isoforms of sulfotransferases in HS biosynthetic activities, while Glce and 2OST each have only a single isoform (Tarja et al., 2005).

At present, pharmaceutical grade heparan sulfate is mainly derived from animal tissues. However, these preparations inevitably include trace contaminants including structurally similar glycosaminoglycans, as well as tissue-derived viruses and prions (Liu et al., 2009). To address these limitations, a chemoenzymatic bioengineered approach has emerged as a promising alternative to animal-sourced heparan sulfate (Fu et al., 2016). This methodology commences with the microbial fermentation of *Escherichia coli* K5 capsular polysaccharide, follows by sequential enzymatic modifications to generate well-defined heparan sulfate structures. Among these steps, Glce protein catalyzes the epimerization of GlcA residues to L-IdoA, a reaction that constitutes a critical step in determining the characteristic structural heterogeneity of heparan sulfate.

Glce is one of the key enzymes in the biosynthesis of heparan sulfate. Targeted disruption of *Glce* gene in mice leads to a structurally aberrant HS lacking of L-IdoA residues, which correlates with a lethal phenotype characterized by renal agenesis, lung defects, and skeletal malformations (Li et al., 2003). Functional investigations demonstrate that *Glce* may affect the proliferation, angiogenesis and metastasis of Ewing sarcoma by altering heparan sulfate biosynthesis and is a potential prognostic indicator in Ewing sarcoma (Wen et al., 2023). Notably, many studies have found that *Glce* gene expression is associated with many tumorigenesis and virus infections (Marques et al., 2021). For example, *Glce* significantly suppresses the proliferative activity of breast cancer cells *in vitro* and tumor growth *in vivo* (Grigorieva et al., 2011; Prudnikova et al., 2014). Cell-surface Glce binds to the porcine deltacoronavirus spike protein, facilitating viral entry (Xiao et al., 2024). Currently, the crystal structure of human Glce protein was reported, these structures elucidate its catalytic mechanism with heparin sulfate. Structure analysis found that the N-terminal β-hairpin and C-terminal α-barrel were major domains to form the dimer structure (Debarnot et al., 2019). However, the molecular basis of N-terminal β-hairpin involvement in functional dimer formation remains to be established.

At present, there is still lack an effective strategy for producing soluble and high-yield Glce protein. Mammalian cells serve as the primary protein expression system to obtain human Glce protein (Cui et al., 2023; Debarnot et al., 2019), but their high operational costs and limited production capacities render large-scale manufacturing technically challenging. In contrast, the *E. coli* expression system offers simplicity, low cost, and high yield, making it an important platform for heterologous expression of membrane proteins and glycosylation modification enzymes. To address this challenge, we utilized the *E. coli* SUMO-fused expression system to express and purify two N-terminal truncated Glce proteins. Static- light and dynamic-light scattering experiments revealed that Glce^167-617^ is consistent with a homogeneous and stable dimer. Additionally, crystallography experiments further demonstrated that Glce^167-617^ protein is dimeric assembly and retains its glycosylation substrate binding sites. This study established a protocol for efficient expression and purification of recombination human Glce^167-617^ in *E. coli*, while confirming its catalytic activity through structural characterization. The crystallographic study was specially designed to compare substrate-binding mechanisms between human and zebrafish Glce orthologs. These founding may facilitate industrial production of Glce protein for the chemoenzymatic synthesize of specific heparin sulfate.

## 2 Materials and methods

### 2.1 Construction of two *Glce* truncation expression plasmids

The *Glce* gene was chemically synthesized according to the codon preference of *Escherichia coli*. The *Glce* (G102-N617) and *Glce* (Y167-N617) gene fragments were amplified from full-length *Glce* cDNA with a 5′ overhang containing the *Eco*RI site and a 3′ overhang containing the *Xho*I site. The PCR products were subsequently inserted into the pET-15b-SUMO vector (Invitrogen) using the *Eco*RI and *Xho*I restriction sites, producing a SUMO and His_6_ tag at the N-terminus. The pET-15b-SUMO-*Glce*^102-617^ and pET-15b-SUMO-*Glce*^167-617^ constructs were confirmed by double restriction enzyme digestion and sequencing.

### 2.2 Expression of Glce truncated proteins in *Escherichia coli*

The recombinant plasmids were transformed into C2566H *E. coli* competent cells (New England Biolabs) for expression of SUMO-Glce^102-617^ and SUMO-Glce^167-617^ fusion proteins. Cells containing the *Glce* gene expression plasmid were initially grown in LA (Luria-Bertani Agar) medium with 100 μg/mL ampicillin at 37°C. Selected colonies were inoculated into Luria Bertani (LB) medium containing 100 μg/mL ampicillin and incubated at 37°C, 180 rpm for overnight. The seed culture was subsequently transferred to LB medium (0.1% inoculation volume) and grown under identical conditions until the cell density reached an OD_600_ of 0.8–1.2. Protein induction was initiated by reducing the temperature was lowered to 20°C, followed by addition of 0.15 mM IPTG and incubation for 16 h at 18°C, 180 rpm.

### 2.3 Purification of Glce truncated proteins

Bacterial cells were collected by centrifugation and resuspended in lysis buffer containing 20 mM Tris–HCl, pH 8.0, 500 mM NaCl, 5 mM imidazole and 5% glycerol (v/v). Following sonication, the lysate was centrifuged at 12,000 rpm for 1 h. The supernatant was loaded onto a Ni^2+^ charged immobilized metal affinity chromatography (IMAC) column (GE Healthcare), followed by sequential washing with lysis buffer and wash buffer [20 mM Tris-HCl, pH 8.0, 500 mM NaCl, 20 mM imidazole and 5% glycerol (v/v)]. Target proteins were eluted with elution buffer [20 mM Tris-HCl, pH 8.0, 500 mM NaCl, 200 mM imidazole and 5% glycerol (v/v)]. Proteolytic cleavage of the N-terminal SUMO and His_6_ tag was removed by incubation the target proteins with SUMO protease at a molar ratio of 250:1 for overnight at 4°C. The tag-free target proteins were diluted in buffer [20 mM Tris–HCl, pH 8.0, 5% glycerol (v/v), 1 mM 1, 4-dithioerythritol (DTT)] and loaded onto a 1 mL HiTrap SP column (GE Healthcare) at 18°C. Bound proteins were eluted using a linear NaCl gradient to 1 M, and the target protein-containing fractions were subsequently diluted in buffer (20 mM Tris-HCl, pH 8.0, 200 mM NaCl, 1 mM DTT). Final the target protein-containing fractions were concentrated to 8 mg/mL using ultrafiltration devices and stored at −80°C.

### 2.4 Static- and dynamic-light scattering

The aggregation state of Glce^167-617^ protein in solution was analyzed using static- and dynamic-light scattering. For static light scattering experiment, which was performed on a Superdex 200 10/300 GL column (GE Healthcare) using a fast protein liquid chromatography (FPLC) system (ÄKTA Purifier, GE Healthcare) at 18°C. The column was equilibrated with buffer (20 mM Tris-HCl, pH 8.0, 200 mM NaCl, 1 mM DTT) and calibrated using gel filtration standard (Bio-Rad) at a flow-rate of 0.2 mL/min. Subsequently, 100 μg of Glce^167-617^ was loaded onto the column, and elution profiles were monitored via UV absorbance at 280 nm. Molecular weight determination of Glce^167-617^ protein was calculated with the fitted standard curve of ln(*M*_w_) = −8.4755*Kav* + 15.145 (*R*^2^ = 0.9841).

Dynamic light scattering (DLS) was performed at room temperature using a DynaPro NanoStar instrument (Wyatt Technology Corporation, United States) with a 20 μL micro-cuvette (Liu et al., 2015). All buffers and samples were filtered with 0.22 μm filter membranes and centrifuged at 12,000 rpm for 30 min. A preparation 2 μM Glce^167-617^ protein was used for the DLS experiment. Following the instrument equilibration, data acquisition durations were maintained between 3 and 5 min, and the collected data were analyzed using Dynamics 7.0 software.

### 2.5 Crystallization

Crystallization experiments were conducted using commercially available crystallization screens with the sitting-drop vapour diffusion method at 20°C. During preliminary screening, crystals of human Glce were grown in drops containing 0.25 μL protein solution (8 mg/mL) and 0.25 μL reservoir solution composed of 0.2 M ammonium sulfate, 0.1 M Tris, pH 6.5–8.5, and 25% (w/v) polyethylene glycol 3,350. Following optimization, rod-shaped crystals were successfully obtained with a reservoir solution containing 0.2 M ammonium sulfate, 0.1 M Tris, pH 8.5, and 22% (w/v) polyethylene glycol 3,350.

### 2.6 Date collection and structure determination

All X-ray diffraction data were collected at beamline BL19U1 of the Shanghai Synchrotron Radiation Facility (SSRF, Shanghai, China) using a Pilatus 6M detector (Dectris) and processed using X-ray Detector Software (XDS) (Kabsch, 2010). Date collection and processing statistics are summarized in Table 1.

**Table 1:** Data collection and refinement statistics.

Data collection	hGlce^167-609^
Wavelength	0.9793
Resolution range (Å)	64.55–2.88 (2.99–2.88)
Space group	*C* 1 2 1
Unit cell a, b, c (Å) *α*, *β*, *γ* (°)	221.14, 77.12, 74.58 90, 95.65, 90
Unique reflections	26,029 (1,316)
Multiplicity	5.1 (5.0)
Completeness (%)	91.0 (92.9)
Mean I/sigma (I)	4.7 (1.3)
Wilson B-factor (Å^2^)	32.89
R-merge	0.39 (1.49)
CC1/2	0.917 (0.376)
Refinement	
R-work/R-free (%)	24.17/28.80
Number of non-hydrogen atoms	6,877
Protein residues	861
RMS (bonds) (Å)	0.008
RMS (angles) (°)	1.48
Ramachandran favored (%)	97.17
Ramachandran allowed (%)	2.72
Ramachandran outliers (%)	0.12
Rotamer outliers (%)	1.75
Average B-factor (Å^2^)	43.91

## 3 Results

### 3.1 Plasmid construction and test expression of SUMO-fused recombinant proteins Glce^102-617^ and Glce^167-617^

To enhance the soluble fragment of human Glce protein and simplify the purification steps, the coding sequence was fused to the C-terminus of the SUMO tag. This fusion provides a simple and rapid purification strategy (Zhou et al., 2020). The recombination protein bearing the SUMO tag could be cleaved by SUMO protease. In addition, a His_6_ tag was incorporated at the N-terminus of the SUMO tag, thereby enabling protein purification via Ni^2+^-IMAC affinity chromatography.

**Figure 1:**
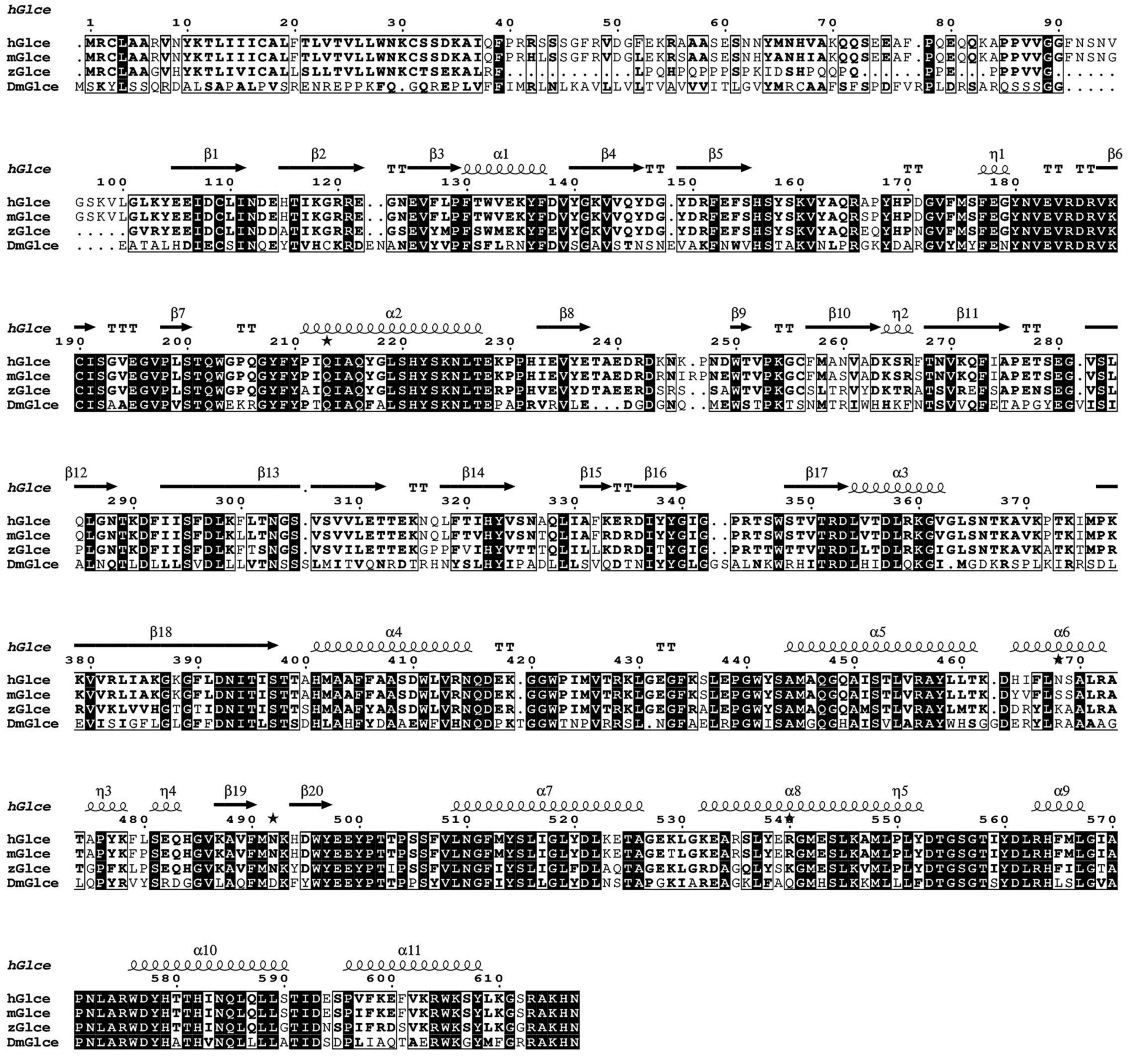
Multiple sequence alignment of hGlce, its close homologs from *Mus musculus* (mGlce) and Zebrafish (zGlce), as well as *Drosophila melanogaster* (DmGlce), was constructed using ClustalW and visualized via ESPript. The identical residues are showed with white color, whereas similar residues are enclosed in boxes. Secondary structure elements of Glce are indicated above the alignment result.

**Figure 2:**
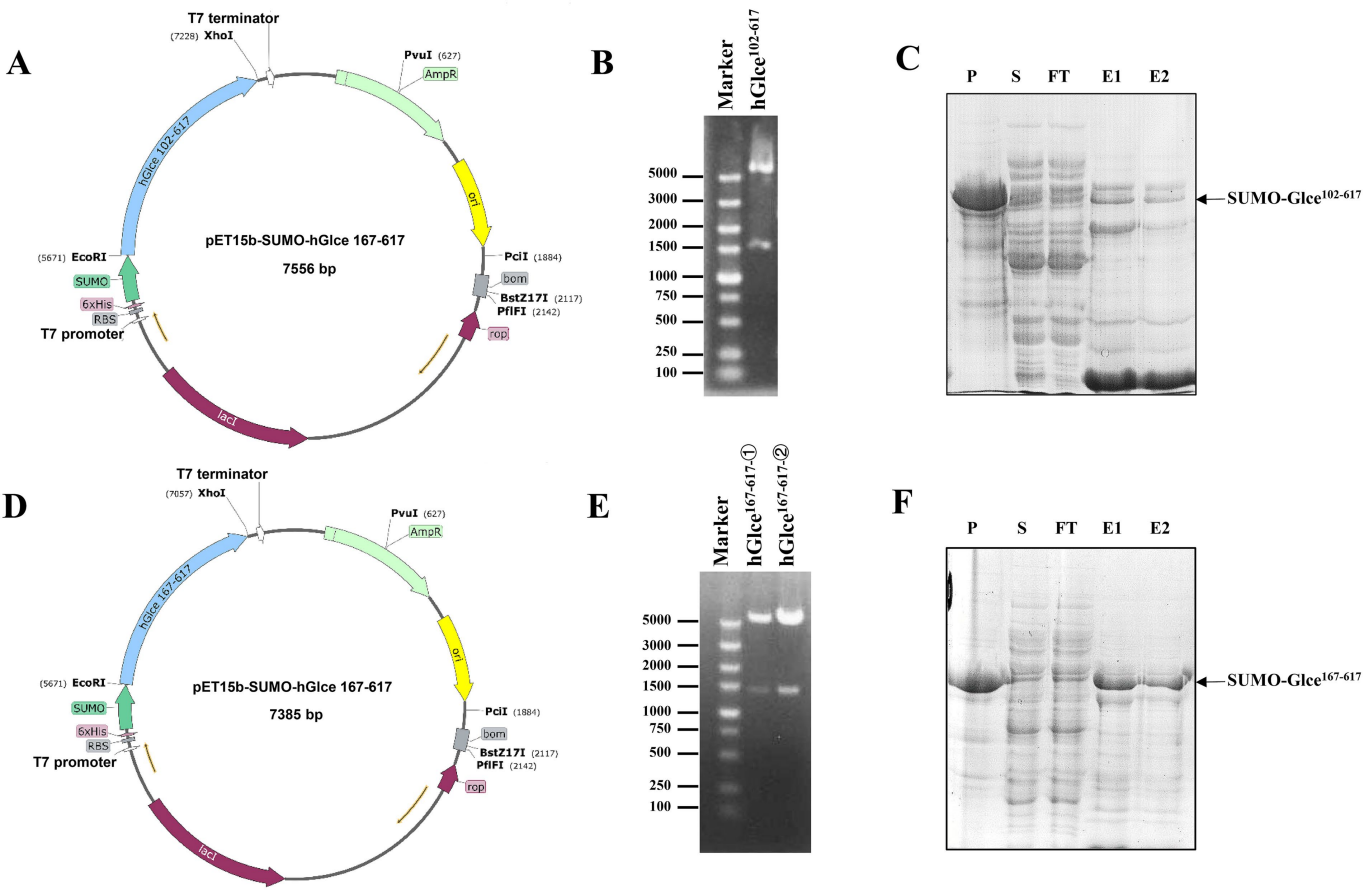
Recombinant plasmids construction and expression of human Glce^102-617^ and Glce^167-617^ fusion protein. **(A)** Schematic of prokaryote expression vector pET-15b-SUMO-*Glce*^102-617^. **(B)** Enzymatic digestion of the recombinant plasmid pET-15b-SUMO-*Glce*^102-617^. **(C)** The SDS-PAGE analysis of SUMO-Glce^102-617^ fusion protein. Arrow shows SUMO-Glce^102-617^ fusion protein. P, precipitate; S, supernatant; FT, flow-through fraction; E1, the first tube of eluted protein; E2, the second tube of eluted protein. **(D)** Schematic of pET-15b-SUMO-*Glce*^167-617^ plasmid. **(E)** Enzymatic digestion of the recombinant plasmid pET-15b-SUMO-*Glce*^167-617^. **(F)** The SDS-PAGE analysis of SUMO-Glce^167-617^ fusion protein. Arrow shows SUMO-Glce^167-617^ fusion protein.

Multiple sequence alignment was conducted to demonstrate the sequence homology among human, mouse, zebrafish and *Drosophila melanogaster*. The alignment illustrated that Glce possesses a highly conserved protein sequence across species (Figure 1). AlphaFold structure prediction showed that the N-terminus of Glce composes one α-helical domain and disordered regions (Supplementary Figure 1). To achieve enhanced expression of Glce protein, two truncated constructs differing in N-terminal truncation positions were generated. In summary, the *Glce^102-617^* and *Glce^167-617^* gene fragments were cloned into the pET-15b-SUMO vector (Figures 2A,D), with construct validation performed through double restriction enzyme and Sanger sequencing (Figures 2B,E).

The *E. coli* C2566H strain was employed as the expression host for recombinant glucuronyl C5-epimerase. An engineered *E. coli* strain harboring *Glce^102-617^* or *Glce^167-617^* gene was cultured at 37°C to reach an OD_600_ value of approximately 0.85. Following induction with 0.15 mM IPTG, SUMO-Glce^102-617^ and SUMO-Glce^167-617^ proteins were induced for efficient overexpression. SDS-PAGE analysis demonstrated that SUMO-Glce^167-617^ protein exhibited a significantly higher expression level compared to SUMO-Glce^102-617^ (Figures 2C,F).

### 3.2 Purification of human Glce^102-617^ and Glce^167-617^ proteins

**Figure 3:**
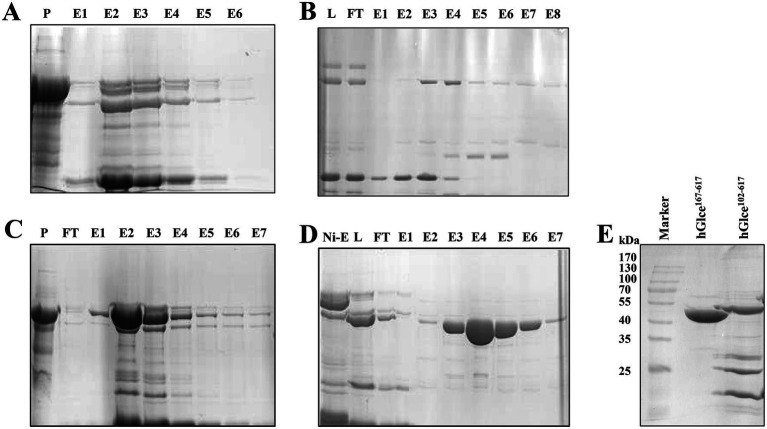
Purification of Glce protein using affinity and ion exchange chromatography. **(A)** SDS-PAGE analysis of SUMO-Glce^102-617^ fusion protein after Ni^2+^ affinity chromatography. P, precipitate; E, the eluted protein. **(B)** Purification of Glce^102-617^ protein after ion exchange chromatography. L: SUMO-Glce^102-617^ was treated with SUMO protease to load onto ion exchange chromatography. **(C)** SDS-PAGE analysis of SUMO-Glce^167-617^ fusion protein after Ni^2+^-IMAC affinity chromatography. **(D)** Purification of Glce^167-617^ protein after ion exchange chromatography. **(E)** The SDS-PAGE analysis of the purified Glce^102-617^ and Glce^167-617^ proteins.

An efficient purification protocol for the hGlce protein was established, demonstrating suitability for biochemical characterization and crystallization. The expressed Glce^102-617^ and Glce^167-617^ proteins underwent purification via a two-step chromatography method comprising Ni^2+^-IMAC column purification followed by HiTrap SP ion exchange chromatography. The first purification step using Ni^2+^-IMAC column eluted SUMO-Glce^102-617^ or SUMO-Glce^167-617^ fused proteins (Figures 3A,C). Subsequently, the eluted proteins underwent SUMO tag cleavage using SUMO protease at 4°C for overnight. The second purification step comprised HiTrap SP ion exchange column with elution conducted through a linear NaCl gradient (0.05–0.5 M) (Figures 3B,D). The purity of purified Glce^167-617^ was exceeded 98%, whereas Glce^102-617^ displayed a purity of approximately 50% (Figure 3E). SDS-PAGE analysis indicated that both proteins migrated with apparent molecular weights slightly smaller than their theoretical molecular weight (Mw). The purified Glce^102-617^ (theoretical Mw: 58.9 kDa) migrated as ~55 kDa protein as determined by SDS-PAGE. Similarly, the purified Glce^167-617^ (theoretical Mw: 52.1 kDa) migrated as ~50 kDa protein by SDS-PAGE. Purification procedures are detailed in Table 2. The yield of Glce^167-617^ was observably higher than that of Glce^102-617^.

**Table 2:** Purify analysis of hGlce^102-617^ and hGlce^167-617^.

Step	Protein name	Total protein^a^ (mg)	Purity^b^ (%)
Cell lysate^c^	hGlce^102-617^	22,930	NA
	hGlce^167-617^	22,190	NA
Supernatant	hGlce^102-617^	4169.645	NA
	hGlce^167-617^	4322.71	NA
Ni NTA column	hGlce^102-617^	6.064	30
	hGlce^167-617^	13.1775	70
HiTrap SP column	hGlce^102-617^	0.66	50
	hGlce^167-617^	5.157	98

a Total protein was determined by NanoDrop ND-2000.

b Protein purity was estimated by SDS-PAGE analysis.

c Lysate was obtained from cells of a 3L culture.

### 3.3 Glce^167-617^ protein act as dimer in solution

**Figure 4:**
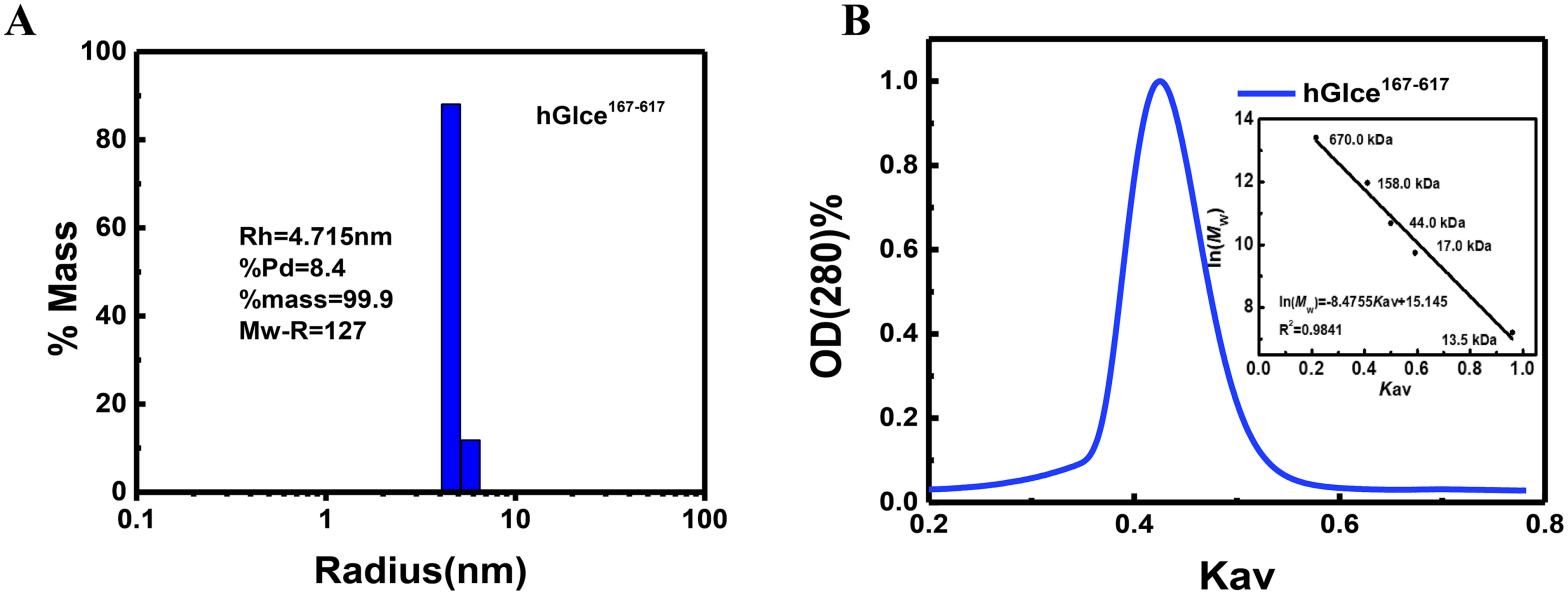
The aggregation state of Glce^167-617^ protein in solution. **(A)** Size distribution histogram of Glce^167-617^ measured by DLS. **(B)** Size exclusion chromatography analysis of Glce^167-617^ by Superdex 200 10/300 GL column. The insert graph displays elution profiles of calibration kit protein on the same column.

The aggregation state of Glce^167-617^ may be disrupted by denaturing conditions during SDS-PAGE. To further determine the homogenicity and aggregation state of Glce^167-617^ in solution, DLS and size exclusion chromatography (SEC) analyses were conducted for Glce^167-617^ characterization. DLS analysis revealed a hydrodynamic radius of 4.715 nm for Glce^167-617^, which corresponded to an estimated molecular weight of 127 kDa (Figure 4A). These results demonstrate that Glce^167-617^ exists as a dimer in solution. Similarly, the SEC analysis revealed a single peak at *K*_av_ = 0.425, yielding an apparent molecular weight of 102.5 kDa based on the calibration equation (Figure 4B).

### 3.4 Crystallization and dimer structure of Glce^167-617^

**Figure 5:**
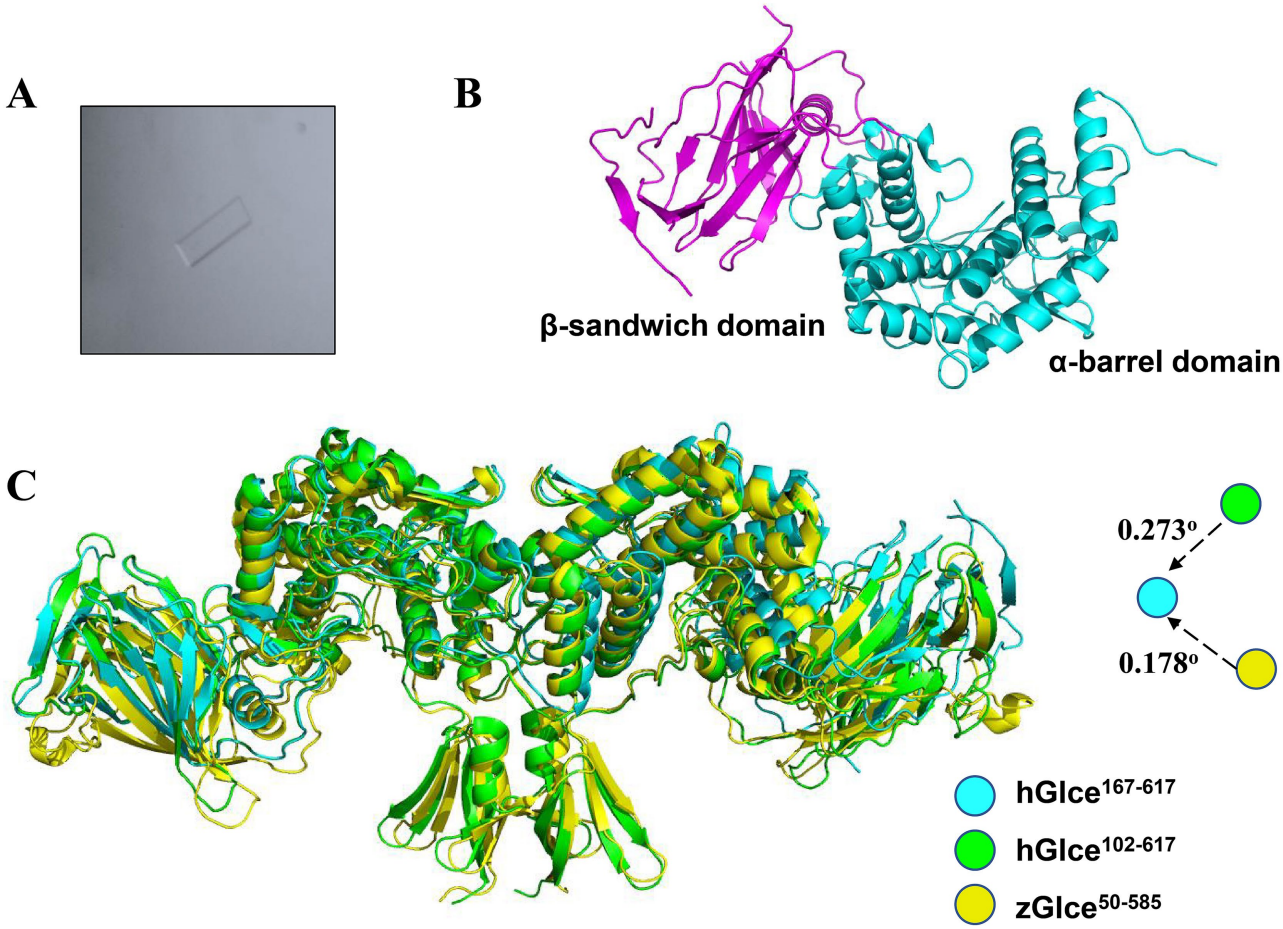
Structure of hGlce^167-617^. **(A)** The micrograph shows typical lamellar crystal of hGlce^167-617^. **(B)** Overall structure of the Glce^167-617^ monomer. Each subunit comprising of β-sandwich and α-barrel domain are colored in magentas and cyan. **(C)** Structure alignment of hGlce^167-617^ to the structure of hGlce^102-617^ and zGlce^50-585^. hGlce^167-617^, hGlce^102-617^ and zGlce^50-585^ are colored in cyan, green, and yellow, respectively.

The pure and homogenous Glce^167-617^ protein was utilized for crystallization trails with different commercial screening kits. We succeeded in screening crystallization conditions, which grew a few single crystals with lamellar structures (Figure 5A). The crystal structure of Glce^167-617^ was determined by molecular replacement and refined to 2.88 Å resolution with R-work/R-free values of 0.24/0.28, respectively. Comprehensive statistics for structure determination and refinement are summarized in Table 1.

Glce^167-617^ crystallized in space group *C* 1 2 1 with two molecules in the asymmetric unit. The Glce^167-617^ structure comprises two domains: an α-barrel domain (residues P167-I233 and D353-N617) and a β-sandwich domain (residues E234-R352). The overall dimeric structure adopts a butterfly-like shape (Figure 5B). Structure analysis reveals a high degree of structural conservation between Glce^167-617^ and both human Glce^102-617^ and zebrafish Glce^50-585^. Structural superposition of hGlce^167-617^ with hGlce^102-617^ and zGlce^50-585^ yielded root-mean-square deviations (RMSD) of 0.273 Å and 0.178 Å, respectively (Figure 5C). As previously reported, dimer formation in Glce involves N-terminal β-hairpin and C-terminal α-barrel domains (Debarnot et al., 2019; Qin et al., 2015). Our results found that deletion of the N-terminal β-hairpin did not disrupt the dimeric architecture in Glce. At the dimerization interface, the C-terminal α-barrel domain forms hydrophobic packing interactions with residues F490, M491, A547, L551, T554, and F565 (Supplementary Figure 2).

### 3.5 hGlce^167–617^ contains conserved substrate binding sites

**Figure 6:**
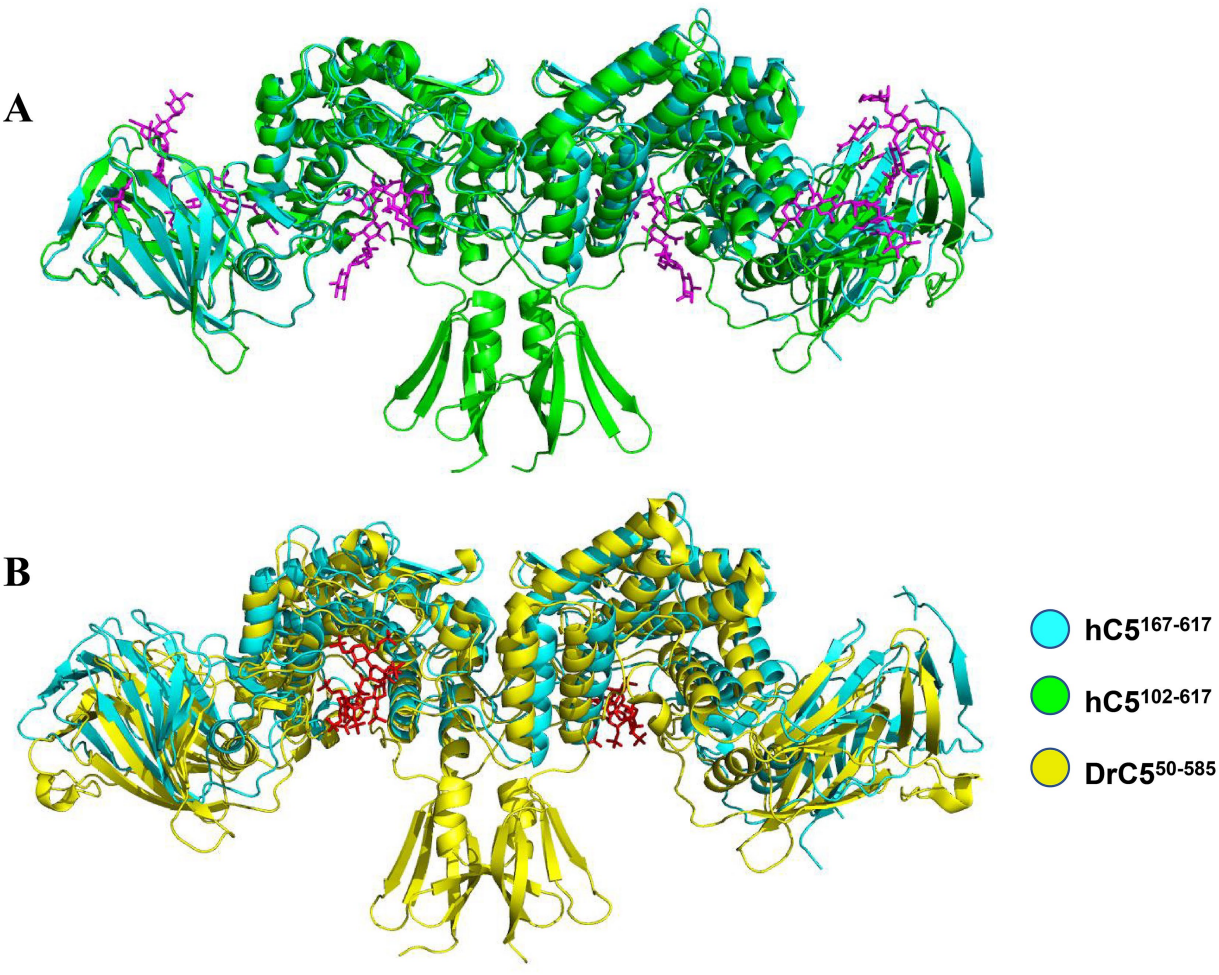
Structure of hGlce^167-617^ contain the conserved substrate binding sites. **(A)** Overall view of substrate binding sites in hGlce^167-617^ and hGlce^102-617^. **(B)** Overall view of (GlcA-GlcNS)_5_ substrate binding sites in hGlce^167-617^ and zGlce^50-585^.

To identify the substrate-binding pocket, structural superposition was performed among three conformations: hGlce^167-617^ apo, hGlce^102-617^ in complex with (GlcA-GlcNS)_5_, and zGlce^50-585^ in complex with heparin. Structural superposition of hGlce^167-617^ apo and the hGlce^102-617^-(GlcA-GlcNS)_5_ complex through alignment of α-barrel domain revealed that the (GlcA-GlcNS)_5_ substrate occupies a cleft between the α-barrel and β-sandwich domains in hGlce^167-617^ (Figure 6A). Similarly, superposition of hGlce^167-617^ apo with zGlce^50-585^-heparin complex via α-barrel domain alignment demonstrated that heparin substrate occupies the same cleft between α-barrel and β-sandwich domains in hGlce^167-617^ (Figure 6B). Structural analysis suggests that hGlce^167-617^ retains evolutionarily conserved substrate-binding sites.

## 4 Discussion

Currently, bioengineered heparin synthesis represents a cost-effective method that has been proposed as an alternative to animal-sourced heparin (Vaidyanathan et al., 2020). Glucuronyl C5-epimerase serves as a key enzyme in the biosynthesis of heparin and heparin sulfate, which plays a vital role in cell–cell and cell–matrix interaction and signaling (Cui et al., 2021; Ori et al., 2008). Human glucuronyl C5-epimerase has been the most extensively characterized because of its functional involvement in tumor suppression (Belyavskaya et al., 2017; Wen et al., 2023; Zhu et al., 2021). However, the recombinant Glce protein exhibits reduced catalytic activity and production yield, which substantially impedes the development of enzymatic methods for producing pharmaceutical-grade heparin.

The *E. coli* expression system was used for the overexpressed human Glce protein in Origami-B cell through the pMalc2x vector, demonstrating epimerase functionality (Li et al., 2010; Préchoux et al., 2015). Although the recombinant Glce protein was successfully expressed and purified, it remained fused to maltose-binding protein (MBP) and contained some GroEL contaminant (Vaidyanathan et al., 2020). Moreover, mammalian HEK293 and HEK-EBNA cells were utilized for human Glce protein expression, resulting in enhanced catalytic activity and production yield (Cui et al., 2023; Debarnot et al., 2019). However, ekaryotic expression systems exhibit high operational costs and low expression efficiency, rendering them unsuitable for large-scale protein expression production. In summary, the *E. coli* expression system provides a streamlined, cost-effective, and scalable platform for high-yield production of target proteins.

In this study, hGlce gene was successfully cloned and overexpressed in the host *E. coli C2566H* strain with the pET-15b-SUMO expression vector. It was previously reported that deletion of 203 amino acids from the N-terminus of the Glce protein resulted complete loss of epimerase activity (Li et al., 2010). In order to enhance Glce protein expression efficiency, two N-terminally truncated constructs (Glce^102-617^ and Glce^167-617^) were generated. The overall expression level and solution fraction of Glce^167-617^ protein demonstrated a remarkable increase compared to full-length Glce, thereby facilitating high-throughput screen of crystallization conditions. Notably, the expression level of Glce^167-617^ protein was considerably improved, which is compared with other different truncations (Li et al., 2010). Hence, this optimization established a feasible strategy for recombinant Glce protein production.

For protein purification, affinity chromatography represents a prevalently adopted technique for target proteins purification, thereby enabling high-yield recovery and exceptional purity from complex protein mixtures (Gomari et al., 2020). The SUMO tag strategy was employed to promote the solubility of Glce protein (Nazir et al., 2024). The purification method of Glce protein involves a two-step chromatographic process that contains affinity purification and ion exchange purification, resulting in highly purified target protein.

The structure and function of Glce are highly conserved across species. Current structural investigations of human and zebrafish Glce have achieved the most comprehensive characterization. Studies have reported that human and zebrafish Glce exist as dimer and function in dimeric form (Debarnot et al., 2019; Qin et al., 2015). Furthermore, *Bermanella marisrubri* Glce adopts dimeric and tetrameric states, with aggregate formation maintaining functional activity (Raedts et al., 2013). Structure analysis found that human and zebrafish Glce protein predominantly adopt tight dimeric configurations through hydrophobic packing interaction and ionic bonds between N-terminal β-hairpin and C-terminal α-barrel domains (Debarnot et al., 2019; Qin et al., 2015). In this study, it was investigated whether Glce dimer remained stable following N-terminal β-hairpin deletion. Initially, DLS and SEC analyses confirmed the dimeric state of Glce^167-617^ protein in solution. Subsequently, crystallographic analysis of Glce^167-617^ showed that the dimeric architecture was preserved after N-terminal β-hairpin removal. Collectively, these findings identify the C-terminal α-barrel domain as the primary dimerization interface. Each Glce dimer contains two active sites that facilitate heparin sulfate modifications.

## 5 Conclusion

In the present study, an *Escherichia coli* expression system has been developed to facilitate efficient expression and purification of Glce protein. SEC and DLS analyses confirmed the stable dimeric configuration of Glce^167-617^ with homogeneous oligomeric state. Moreover, the crystal structure of Glce^167-617^ was resolved at 2.88 Å resolution, revealing a complete dimeric architecture that preserves the GlcA epimerization site. Collectively, these findings establish a simple and efficient approach for producing active Glce protein, thereby facilitating the biosynthesis of heparin and heparan sulfate *in vitro*.

## Data Availability

The datasets presented in this study can be found in online repositories. The names of the repository/repositories and accession number(s) can be found in the article/Supplementary material.
