# Supplementary material for: A high effective expression of human D-glucuronyl C5-epimerase with dimer structure in Escherichia coli
Source: Front Microbiol. 2025 Jul 31;16:1641598. doi: 10.3389/fmicb.2025.1641598 (PMC12350261; doi:10.3389/fmicb.2025.1641598)
Supplement: Supplementary file 1 [file Supplementary_file_1.docx]

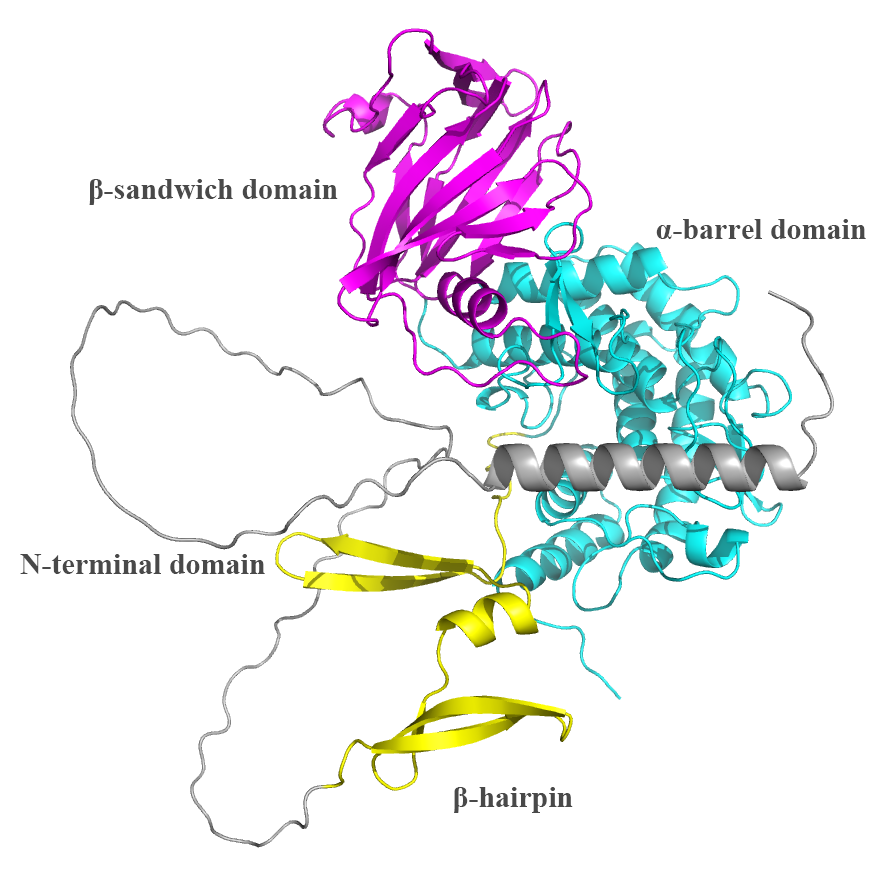


**Supplemental Figure 1.** The predicted structure of hGlce by AlphaFold. N-terminal subdomain is colored by gray; β-hairpin is colored by yellow; β-sandwich domain is colored by cyan; C-terminal α-barrel domain is colored by green.


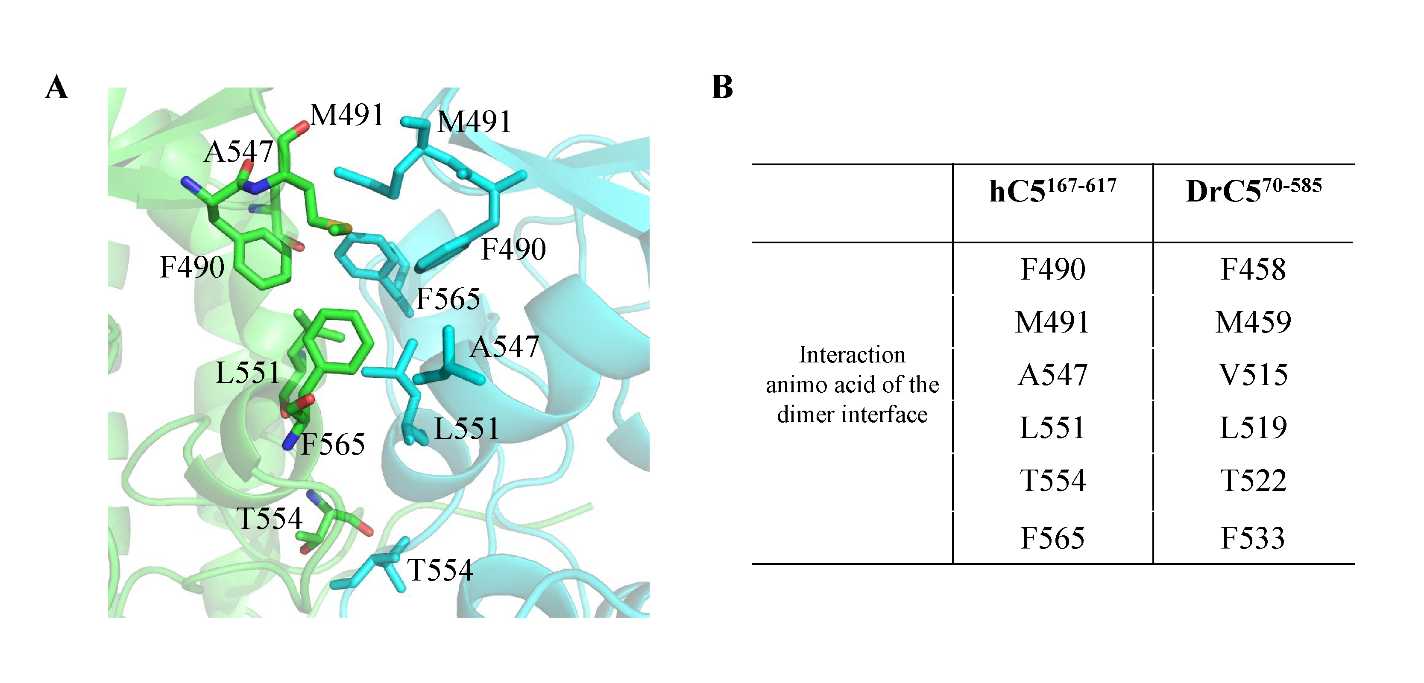


**Supplemental Figure 2.** The dimer interface at C-terminal α-helical domain. (A) Detailed view of hydrophobic interaction residues. (B) Residues involved in the formation of C-terminal α-helical domain in hGlce and zGlce.
